# Preoperative application of carbon nanoparticles in transoral endoscopic thyroidectomy vestibular approach for papillary thyroid cancer

**DOI:** 10.3389/fonc.2023.1120411

**Published:** 2023-03-09

**Authors:** Yonghui Wang, Li Zhang, Jinning Huang, Liquan Wang

**Affiliations:** Department of Thyroid and Breast Surgery, Weifang People’s Hospital, Weifang, China

**Keywords:** preoperative injection, papillary thyroid cancer, transoral endoscopic thyroidectomy vestibular approach, carbon nanoparticles, intraopertive injection

## Abstract

**Background:**

Carbon nanoparticles (CNs) have been widely used in the protection of the parathyroid gland and act as a tracer agent in central lymph node dissection. However, the right time for CN injection has not been well illustrated in the transoral endoscopic thyroidectomy vestibular approach (TOETVA). The purpose of this study was to evaluate the safety and feasibility of the preoperative injection of CNs in TOETVA for papillary thyroid cancer.

**Methods:**

From October 2021 to October 2022, a total of 53 consecutive patients with PTC were retrospectively analyzed. All patients underwent unilateral thyroidectomy *via* the TOETVA. The patients were divided into the preoperative group (*n* = 28) and the intraoperative group (*n* = 25) according to CN injection time. In the preoperative group, 0.2 ml of CNs were injected into the thyroid lobules with malignant nodules 1 h before surgery. The numbers of total central lymph node (CLN) and metastatic central lymph node (CLNM), parathyroid autotransplantation, accidental removal of the parathyroid, and the parathyroid hormone level were recorded and analyzed.

**Results:**

The leakage of CNs happened more frequently in the intraoperative group than in the preoperative group (*P* = 0.002). The mean number of retrieved CLN and CLNM was similar in the preoperative group and the intraoperative group. In parathyroid protection, more parathyroid was discovered in the preoperative group than in the intraoperative group (1.57 ± 0.54 *vs*. 1.47 ± 0.50, *P* = 0.002), but less parathyroid autotransplantation (*P* = 0.004) and accidental removal of the parathyroid (*P* = 0.036) were discovered in the preoperative group. However, the PTH level between the two groups was similar after the first day and the first month.

**Conclusion:**

The preoperative injection of CNs is a safe and effective method to protect the parathyroid glands (PGs) in patients with PTC undergoing TOETVA. However, the value of preoperative injection of CNs in TOETVA for central lymph node dissection needs to be further studied.

## Introduction

Papillary thyroid carcinoma is the most common endocrine tumor worldwide (1, 2). The transoral endoscopic thyroidectomy vestibular approach (TOETVA) provides easy access to the thyroid and central compartment and avoids skin scarring during thyroid surgery, which makes it a widely popular option in thyroidectomy (3).

Carbon nanoparticles (CNs) act as a novel lymph node tracer and have been used in the surgery of stomach carcinoma, breast cancer, and thyroid cancer (4, 5). In thyroidectomy, CNs are usually injected into the thyroid gland during operation and stain the gland and lymph node black, which facilitated the surgeons to identify the parathyroid and guide them to dissect the lymph nodes (6). However, CNs might leak out of the thyroid and stain the surrounding tissues during the intraoperative injection. Recent studies have shown that the preoperative injection of CNs is safe and feasible in thyroidectomy *via* traditional open thyroid surgery and bilateral axillo-breast approach robotic thyroidectomy (7–9). However, the effectiveness of the preoperative injection of CNs has not been well illustrated in TOETVA. Therefore, the purpose of this study was to discover the appropriate time to inject CNs in TOETVA.

## Materials and methods

### Patients

From October 2021 to October 2022, 53 consecutive adult papillary thyroid cancer (PTC) patients who underwent TOETVA with central lymph node dissection at the Department of Thyroid and Breast Surgery, Weifang People’s Hospital, were retrospectively enrolled. Patients were divided into the preoperative CN injection group (preoperative group, *n* = 28) and the intraoperative CN injection group (intraoperative group, *n* = 25).

The inclusion criteria were as follows: patients with cosmetic requirements, patients with the longest diameter of tumor less than 20 mm, and patients with postoperative pathologically confirmed PTC.

The exclusion criteria include patients with a history of thyroid surgery or neck radiotherapy, patients younger than 18 years of age, and patients with postoperative pathology suggesting other types of tumors, such as benign tumor, follicular cancer, and medullary or undifferentiated cancer.

All the TOETVA was performed by the same professional thyroid surgeon (Yonghui Wang).

### CN injection before the operation

**Figure 1:**
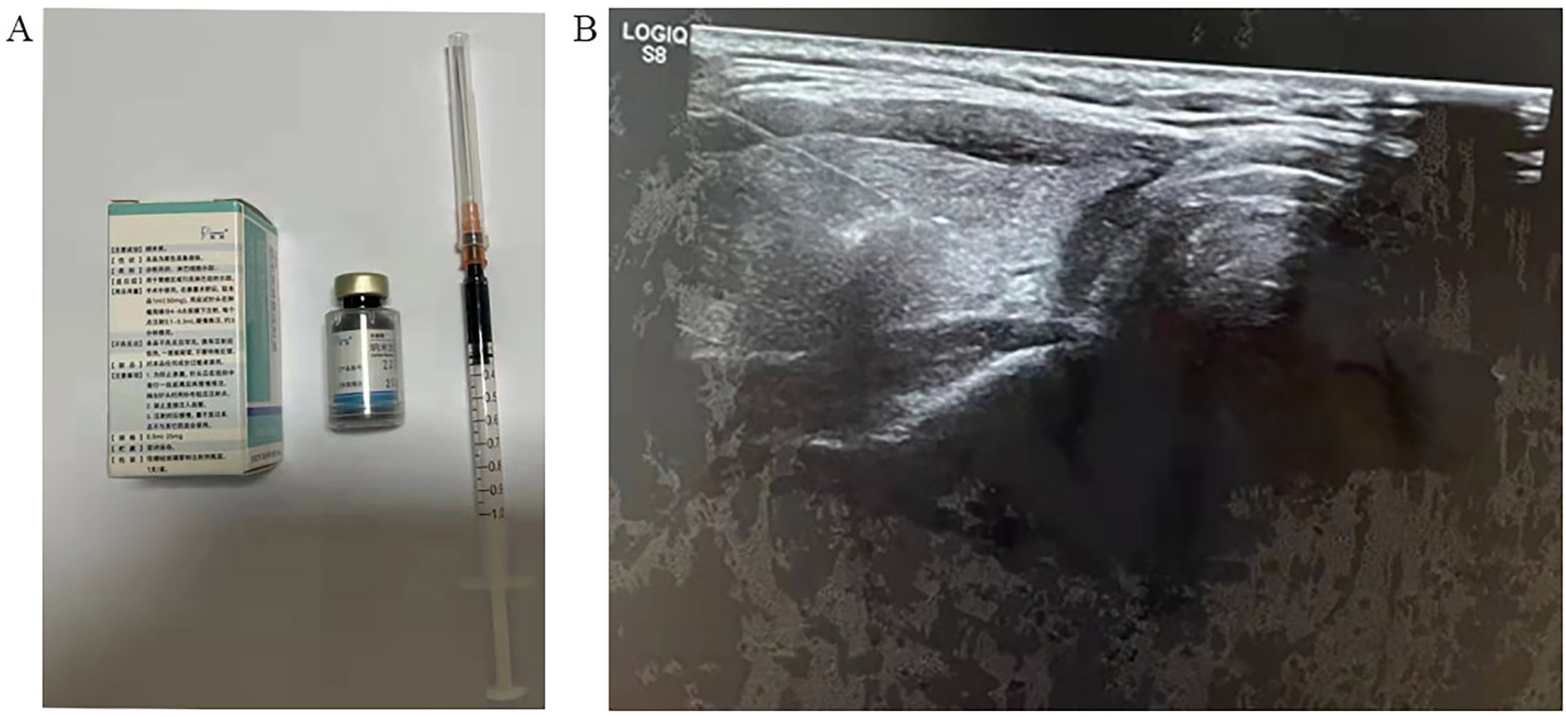
Carbon nanoparticle injection. **(A)** Preparation of carbon nanoparticles. **(B)** The ultrasound-guided carbon nanoparticle injection.

Ultrasound-guided injection of CNs was performed 1 h before surgery in the preoperative group. Carbon nanoparticles (0.5 ml per ampoule, Chongqing LaiMei Pharmaceutical Co., Ltd., Chongqing, China) were used in this study. The special procedures were as follows: 0.2 ml of CNs were extracted with a 1-ml syringe, then a new needle was used, and the air inside it was expelled (**Figure 1A**). Patients were in the position of high shoulder with a pad, and disinfection around the puncture point was prepared before the injection of CNs. We chose a safe and short way to avoid puncturing into the vessels, tumor, and nerves under ultrasound guidance. A volume of 0.2 ml of CNs was injected into the normal thyroid gland tissues, and the needle was gently withdrawn with negative pressure (**Figure 1B**). After the injection, 1 h of observation was needed, so that timely disposal could be arranged once the patients felt unwell. Preoperative injection of CNs was conducted under ultrasound guidance by an experienced surgeon (Liquan Wang) 1 h before the operation.

### Injection of CNs during surgery

The CNs could not be injected under direct vision due to the endoscopic surgery. A percutaneous puncture was made by locating the CN puncture site on the ceiling skin of the working space. CNs (0.2 ml) were injected into the normal thyroid tissue by using a syringe (1 ml). Back-drawing should be performed during injection to avoid mistakenly injecting into the blood vessel. The needle puncture site was gently pressed using gauze for 10 min.

### Surgical procedure

All the patients were diagnosed as unilateral PTC by preoperative fine-needle aspiration, and unilateral thyroid gland dissection was first performed followed by ipsilateral central neck lymph node dissection. The TOETVA operative procedures have been previously described (10).

### Monitoring indicators

The general characteristics, complications of CNs (pain, hematoma, and CN leakage), pathological examinations, and parathyroid gland (PG)-related parameters [mean number of PGs *in situ*, mean number of PG autotransplantation, and postoperative parathyroid hormone level (PTH)] were collected and analyzed.

The level of PTH would be tested at three time points, i.e., during preoperation and 1 day and 1 month after the operation. Hypoparathyroidism is defined as a decline in serum PTH below 15 pg/ml. The patient was considered to have permanent hypoparathyroidism when the serum PTH level at 3 months after surgery was below 1.3 mmol/L.

### Statistical analysis

Continuous variables were presented as mean ± standard deviation and compared using independent samples *t*-tests. Chi-square tests were performed to analyze categorical data. Statistical analysis was performed using SPSS 17.0, and a value of *P <*0.05 was considered statistically significant.

## Results

### Patients’ characteristics

**Figure 2:**
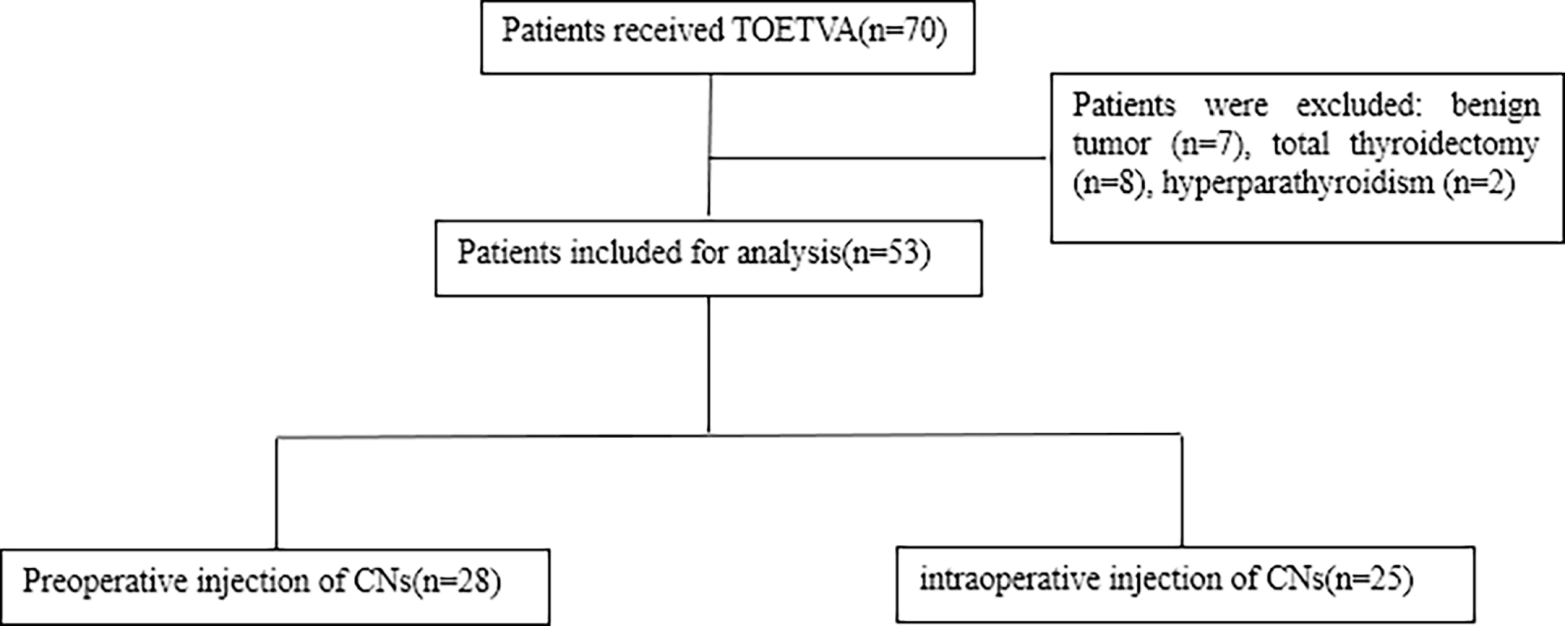
A CONSORT diagram showing the patient inclusion and exclusion criteria. TOETVA, transoral endoscopic thyroidectomy vestibular approach; CNs, carbon nanoparticles.

The process of patient selection based on the inclusion and exclusion criteria is shown in **Figure 2**. TOETVA was performed on a total of 70 consecutive patients. Fifty-three patients (28 patients in the preoperative group and 25 patients in the intraoperative group) were eligible and 17 patients were excluded. Clinical data were retrospectively collated from 53 patients. The ages of the patients ranged from 20 to 60 years, with a median age of 39 years. All patients underwent endoscopic unilateral thyroidectomy with central lymph node dissection (CND) (level VI), and no patient was converted to open thyroidectomy. PTC was confirmed by postoperative pathology for all patients. The clinicopathological characteristics of the patients enrolled in this study are summarized in **Table 1**. There was no significant difference between the preoperative group and the intraoperative group in terms of age, sex, Hashimoto’s thyroiditis, extrathyroid extension, tumor size, and preoperative PTH.

**Table 1:** Patients’ clinical characteristics in the preoperative group and the intraoperative group.

Characteristics	Preoperative group (*n* = 28)	Intraoperative group (*n* = 25)	*P*-value
Age	40.14 ± 1.65	38.60 ± 2.41	0.593
Sex (male/female)			0.883
Male	3	3	
Female	25	22	
Extrathyroid extension			0.184
No	14	8	
Yes	14	17	
Hashimoto’s thyroiditis			0.823
No	6	6	
Yes	22	19	
Tumor size (mm)	0.62 ± 0.45	0.55 ± 0.29	0.928
PTH (preoperative)	40.75 ± 2.47	38.37 ± 2.15	0.478

PTH, parathyroid hormone.

### Safety and tolerance of the CN injection

The injection time was approximately 2 min. No patient in the preoperative group has obvious systemic toxicity. In addition, there was no intolerable pain, bleeding, or hematoma during the injection procedure. One patient had skin marking at the puncture site after injection at the early stage, and we put the drainage tube in the marking site during the operation. CN leakage happened in two patients in the preoperative group and in 11 patients in the intraoperative group. The CN leakage rate was higher in the intraoperative group than in the preoperative group (*P* = 0.002) (**Table 2**).

**Table 2:** Central lymph node dissection and CN leakage in the preoperative group and the intraoperative group.

Variables	Preoperative group (*n* = 28)	Intraoperative group (*n* = 25)	*P*-value
Number of CLN	4.50 ± 0.49	5.00 ± 0.56	0.502
Metastatic CLN	0.75 ± 0.24	0.60 ± 0.19	0.775
CN leakage			0.002
No	2	11	
Yes	26	14	

CLN, central lymph node; CLNM, central lymph node metastasis.

### Lymph node dissection

As shown in **Table 2**, a total of 124 and 125 lymph nodes were dissected in the preoperative group and the intraoperative group, respectively. In the preoperative group, there were 1–12 lymph nodes per case with an average of 4.50 ± 0.49 lymph nodes per case, and 20 lymph nodes had metastases. In the intraoperative group, there were 1–13 lymph nodes per case with an average of 5.00 ± 0.56 lymph nodes per case, and 15 lymph nodes had metastases. However, there was no statistical difference in both total CLN (*P* = 0.502) and CLNM (*P* = 0.775) between the preoperative group and the intraoperative group.

### Identification and protection of the parathyroid glands during the operation

**Figure 3:**
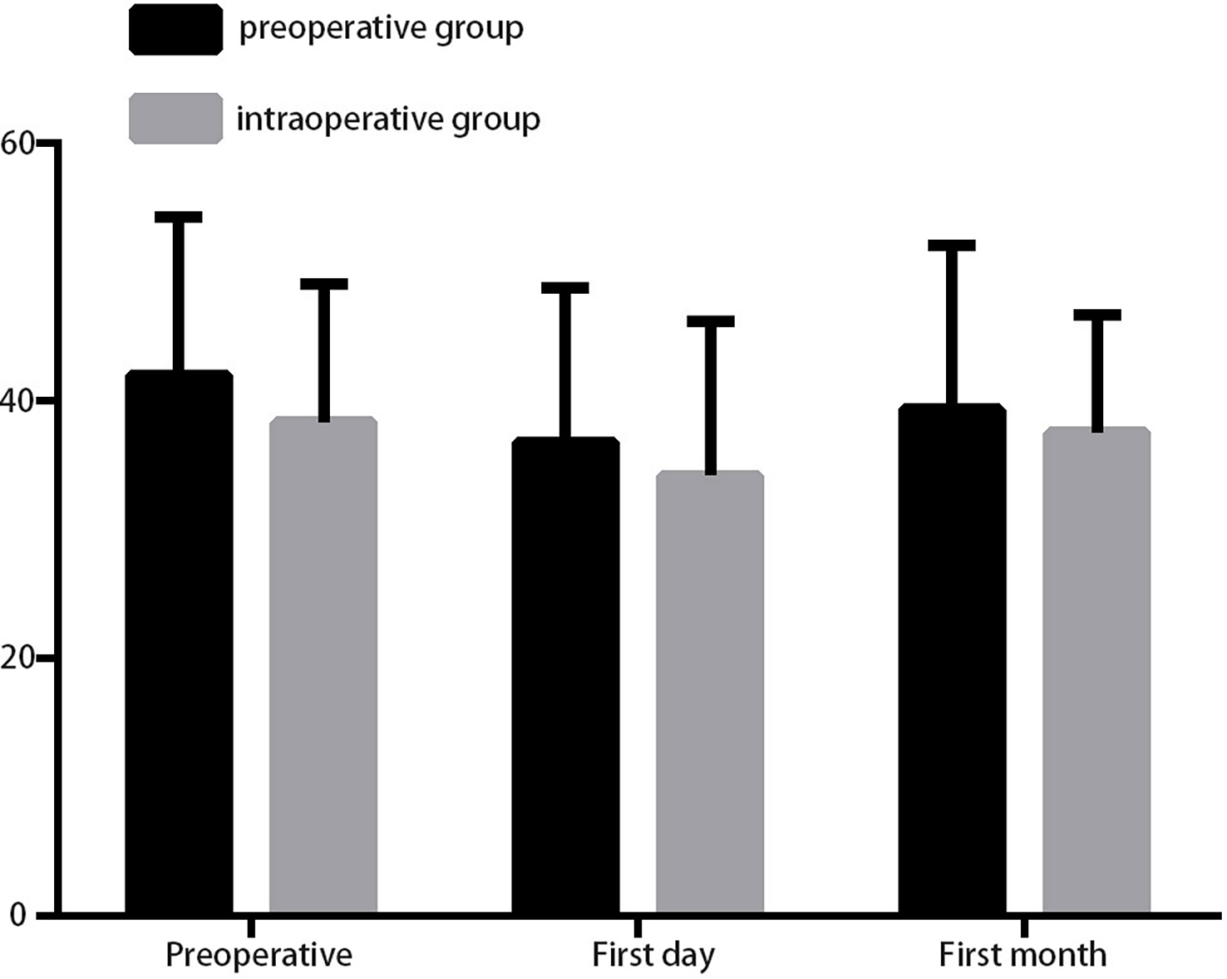
The parathyroid hormone was detected during preoperation and on the first day and first month after surgery.

The baseline of preoperative PTH was similar between the preoperative group and the intraoperative group (40.75 ± 2.47 *vs*. 38.37 ± 2.15, *P* = 0.478) (**Figure 3**). Although the postoperative PTH level dropped on the first day, no significant difference was found between the preoperative group and the intraoperative group, and it recovered to preoperative levels on the first month (**Figure 3**). Pathological results showed that one incident of accidental removal of PG occurred in the preoperative group, whereas seven instances of PG removal occurred in the control group (**Table 3**), which means that there was more frequent accidental PG removal in the intraoperative group (*P* = 0.036). In addition, there was a low ratio of PG autotransplantation in the preoperative group than in the intraoperative group (*P* = 0.004) (**Table 3**).

**Table 3:** Preserved PG *in situ*, PG autotransplantation, and accidental PG removal between the preoperative group and the intraoperative group.

Variables	Preoperative group (*n* = 28)	Intraoperative group (*n* = 25)	*P*-value
Identification of PG	50 (1.57 ± 0.54)	45 (1.47 ± 0.50)	0.002
Autotransplantation of the parathyroid			0.004
No	26	10	
Yes	2	15	
Accidental PG removal			0.036
No	27	18	
Yes	1	7	

PG, parathyroid gland.

## Discussion

CNs, with a diameter of 150 nm, can pass through the lymphatic vessels and accumulate in the lymph nodes and stain them (11). CNs have been widely used as a tracer for lymph node dissection in thyroid surgery. As they do not enter the blood capillaries, the other function of CNs is to discover the parathyroid which is not changed and left unstained unlike the thyroid and lymph nodes (11). More surgeons usually injected CNs during the operation and noticed the phenomenon of CNs leaking out during surgery which stained the surrounding tissue and affected the recognition of the parathyroid (12). Recent studies have shown that the preoperative use of CNs has more advantage in the protection of the parathyroid and in central lymph node dissection in open thyroidectomy and bilateral axillo-breast approach robotic thyroidectomy (7, 8). However, the right time for CN injection has not been well illustrated in TOETVA.

Similar to previous studies with the preoperative injection of CNs, this study showed a few complications of CNs, which is also consistent with the intraoperative injection of CNs in other studies (7, 8). Skin staining after injection occurred in one patient during the early stage of our study, and this adverse event could be avoided by using a new needle before the injection. In addition, the leakage of CNs occurred in two patients in the preoperative group, which is much lesser than in the intraoperative group. There are some reasons for the less chance of leakage in the preoperative injection of CNs. Firstly, the capsule of the thyroid has not been destroyed before the surgery which can prevent the CNs from leaking. Secondly, the trap muscle is still in contact with the capsule and could compress the injection site to prevent leaking.

Due to less scarring in the neck, endoscopic thyroid surgery techniques including the transoral approach and the bilateral axillo-breast approach have been used all over the world (13). The transoral approach does not cause scarring in the skin and requires a smaller subcutaneous flap elevation than the bilateral axillo-breast approach; therefore, TOETVA attracted the attention of both surgeons and patients (3). A previous study showed that central lymph node metastasis occurred in nearly half of the patients and prophylactic central lymph node dissection was performed to reduce its recurrence (14). However, the central lymph node dissection has enhanced the injury of the parathyroid and induced the occurrence of postoperative hypoparathyroidism (14).

Injury to the parathyroid would lead to hypocalcemia, which is often caused by the accidental removal of the parathyroid gland or damage to the glandular blood supply. As a result of the surgeons’ awareness of parathyroid protection and operative skills, the parathyroid injury rate has been reduced. However, the postoperative hypocalcemia rate is still up to 0.3%–49% (14). Therefore, protecting the blood supply and parathyroid is of primary importance in thyroid surgery. In terms of parathyroid protection, there was a low ratio of autotransplantation and accidental thyroid dissection in the preoperative group than in the intraoperative group. The plausible reason was that there was more leaking of CNs in the intraoperative group than in the preoperative group, which darkened the parathyroid and made it hard to distinguish the parathyroid from the adipose tissues. In our study, the accidental removal of the parathyroid happened in seven patients, and all of them had CN leakage. Therefore, it is important to prevent CN leakage by preoperative injection. Most people have four parathyroids. Once one or two parathyroids are injured in the operation, the other parathyroid could enhance the secretion function and act as a substitute for the injured parathyroid. Therefore, the PTH level was not significantly different between the two groups on day 1 in this study. This result was special for total thyroidectomy and bilateral central lymph node dissection which had more possibility to damage all the four parathyroids and develop permanent hypoparathyroidism.

Previous studies showed that CNs could help detect the lymph nodes and increase the discovery of metastatic lymph nodes. However, consistent with the recent study, the time of CN injection could not help in discovering more lymph nodes and metastatic lymph nodes, and this can be attributed to the fact that skillful surgeons could perform central neck dissection according to the requirements of the guidelines.

However, there are some limitations in this study. Firstly, this study was not a prospective randomized control one, which means that the evidence provided by the study was not as powerful as that of a multicentric pragmatic randomized control clinical trial. Secondly, the number of patients in our study was small, and unilateral thyroidectomy was performed which might influence temporary and permanent hypoparathyroidism. In addition, the time of preoperative injection was only 1 h before surgery. In a future study, we might consider more time points such as immediately after anesthesia which might alleviate anxiety.

## Data availability statement

The original contributions presented in the study are included in the article/supplementary material, further inquiries can be directed to the corresponding author/s.

## Ethics statement

The studies involving human participants were reviewed and approved by ethics committee of WeiFang people’s hospital. The patients/participants provided their written informed consent to participate in this study.

## Author contributions

All authors listed have made a substantial, direct, and intellectual contribution to the work and approved it for publication.
